# From SARS to COVID-19: A previously unknown SARS- related coronavirus (SARS-CoV-2) of pandemic potential infecting humans – Call for a One Health approach

**DOI:** 10.1016/j.onehlt.2020.100124

**Published:** 2020-02-24

**Authors:** Mohamed E. El Zowalaty, Josef D. Järhult

**Affiliations:** a Zoonosis Science Center, Department of Medical Biochemistry and Microbiology, Uppsala University, Uppsala, Sweden; b Infectious Diseases and Anti-Infective Therapy Research Group, College of Pharmacy and Sharjah Medical Research Institute, University of Sharjah, United Arab Emirates; c Zoonosis Science Center, Department of Medical Sciences, Uppsala University, Uppsala, Sweden

## Abstract

Human coronaviruses continue to pose a threat to human health. The emergence of severe acute respiratory syndrome coronavirus 2 (SARS-CoV-2) in December 2019 which causes coronavirus disease-2019 (COVID-19), an acute respiratory disease marked the third introduction of a highly pathogenic coronavirus into the human population in the twenty-first century. This recent emergence of a previously unknown coronavirus in China leads to huge impacts on humans globally. Covid-19 is a challenge to global public health. Here, we discuss the COVID-19 outbreak in a one health context, highlighting the need for the implementation of one health measures and practices to improve human health and reduce the emergence of pandemic viruses.

Today, the world faces many complex problems, such as emerging infections, that a single discipline, institution or country cannot respond to alone. The human pulmonary system is vulnerable to infections due to contact-based inoculation of infectious material in droplets through the eyes, nose, or mouth, and airborne transmission is effective as seen e.g. in the plethora of viral respiratory diseases affecting individuals of all age groups [1].Thus, respiratory viruses pose a continuous pandemic threat, of which coronaviruses and specifically the genus *Betacoronavirus* in the family *Coronaviridae* is a subset. During the past decades, humans have been challenged with a number of emerging viral respiratory infections with pandemic potential including the Severe Acute Respiratory Syndrome coronavirus (SARS-CoV) which emerged in China in 2002 [2,3], swine-origin pandemic (H1N1) influenza A virus which emerged in Mexico in 2009 [4] and the Middle East Respiratory Syndrome coronavirus (MERS-CoV) which emerged in Saudi Arabia in 2012 [5].

Coronaviruses represent a continuous pandemic threat; humans have experienced two coronavirus-related health security crises since 2003. In December 2019, a previously unknown coronavirus was discovered in Wuhan city in China [6,7] which initially resulted in a cluster of viral pneumonia cases [8] and later caused an escalating number of reported infections in humans in China and globally [[9], [10], [11]]. The mortality of the emerging coronavirus of 2019 seems mainly to be caused by acute respiratory distress syndrome (ARDS) [12] which may be associated with comorbidities and followed by multiple organ failure leading to death [13]. It is probable that this 2019 coronavirus outbreak is not the last one due to a coronavirus. A provisional name was initially given to this coronavirus as 2019-novel coronavirus (2019-nCoV) and was recently designated as severe acute respiratory syndrome coronavirus 2 (SARS-CoV-2) by the *Coronaviridae* Study Group of the International Committee on Taxonomy of Viruses (ICTV) [14]. The WHO announced that the disease caused by the SARS-CoV-2 is referred to as coronavirus disease-2019 (COVID-19) [15].

Despite recent efforts in basic and translational influenza and coronavirus research, there is still no vaccine against coronaviruses for use in humans (this includes SARS and MERS) [[16], [17], [18], [19]]. In addition, there is yet no universal influenza vaccine available against all influenza virus subtypes and hence seasonal influenza vaccines have to be updated annually and vaccines for pandemic preparedness are a challenge [[20], [21], [22], [23], [24], [25]]. The lack of preventive vaccines for clinical use in humans against such viruses makes emerging influenza and coronaviruses a serious global threat.

Since the emergence of SARS-CoV and MERS-CoV, bats have been the suspect of harbouring emerging viruses. Several studies have recently reported the detection of coronaviruses of pandemic potential [26,27]. Genetic evolutionary analysis of SARS-CoV-2 revealed that this virus is genetically related to two bat coronaviruses [7,28]. Contrary to SARS-CoV and MERS-CoV, human infections due to SARS-CoV-2 have been reported to a quite large extent outside the epicentre of the infection. The numbers of infections due to SARS-CoV-2 continued to grow since its emergence till January 31 (Fig. 1), and as of the date of this publication, the virus has caused more than 80,000 confirmed and reported cases in humans globally [11,29]. Through rapid and frequent international air travel, infections due to SARS-CoV-2 have spread to over 36 countries around the world causing more than 2600 deaths including deaths outside China in Japan, Taiwan, the Philippines, Iran, South Korea, Italy and France have been reported as of 24 February 2020 [9,29]. The epidemiological data available at the time of this publication are summarized in Fig. 2 [10]. Infections due to SARS-CoV-2 are yet unreported at the time of this publication in South American countries. Except for Egypt where one travel-related case was reported on 12 February 2020, COVID-19 infections are not yet reported elsewhere in Africa. As of the date of this report, approximately 97% (*n* = 77,150) of infections were reported in China[29], however this number of infections may not reflect the true situation in China since additional cases may not have been reported to health authorities at the time of the outbreak. As of 24 February, COVID-19 cases have been reported outside of mainland China (2374 cases), where there have been 44 infections in North America (35 in USA, 9 in Canada), 178 infections in Europe (16 in Germany, 12 in France, 13 in UK, 215 in Italy, 2 Spain, and 1 in each of Belgium, Finland, Sweden), 22 in Australia. COVID-19 infections in Asia excluding mainland China were reported as of the date of this publication from Japan (154), 691 on international conveyance Japan, Thailand (35), Singapore (89), Hong Kong (79), South Korea (833), Iran (61)Taiwan (28), Malaysia (22), Vietnam (16), United Arab Emirates (13), Macau (10), India (3), the Philippines (3), Russia (2), Oman (2), Kuwait (1), Bahrain (1), Afghanistan (1), Lebanon (1), Israel (1), Cambodia (1), Nepal (1), and Sri Lanka (1) [10,29].

**Fig. 1:**
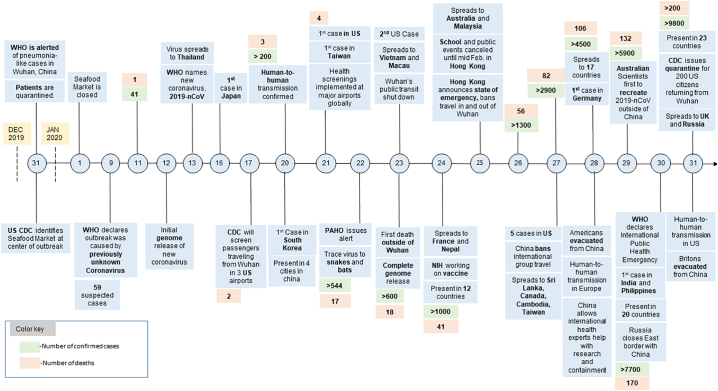
Timeline of COVID-19 cases worldwide since 31 December 2019 until 31 January 2020. (The figure was reproduced with permission from Kara Kochek of Duke One Health Research Team, Duke University, North Carolina, USA).

**Fig. 2:**
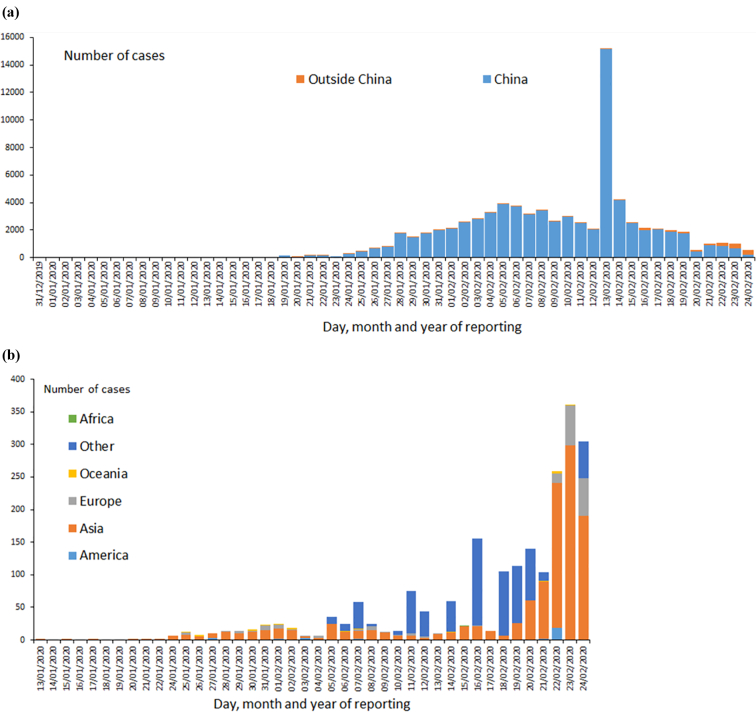
Distribution of laboratory confirmed COVID-19 cases (a) worldwide (b) continent (except China) as of 24 February 2020. (Reproduced from [10].

The case fatality rate is calculated by dividing the number of known deaths by the number of confirmed cases. The resulting number, however, does not represent the true case fatality rate and might be off by orders of magnitude [30]. The true case fatality rate is unknown at this stage of the outbreak, and its precise estimate is impossible at present [30,31]. The current estimates of case fatality rate of SARS-CoV-2 at any time point of analysis should be interpreted with caution since the outcome of the emerging COVID-19 is yet unknown. There are 33 fatalities reported outside China as of the date of this report. On the contrary, the case fatality rate with SARS was 10% and the US identified eight patients with no fatalities. For MERS, the fatality is 35% and the US identified two patients with no fatalities, and sporadic MERS cases are being reported mainly from the Arabian Peninsula till this day. As a comparison, Influenza A virus infections in the current season (2018–2019) led to an estimated 490,561 hospitalizations and 34,157 deaths in the US [32]. Although the numbers for Influenza A virus infections are not obtained in the same way as for SARS-CoV-2, SARS-CoV, and MERS-CoV, and thus are not directly comparable, they still serve as an important reminder of the large numbers of deaths a ‘low-mortality’ infection can cause when widespread in the community.

Globally, the clinical picture in humans infected with SARS-CoV-2 have ranged from mild (no or minor) to severe signs and symptoms including death. It was reported that the first instance of COVID-19 related pneumonia cases, whether linked to the Huanan Seafood Market or not, occurred between 6 and 15 December 2019 [33]. Another study reported the onset of pneumonia cases related to COVID-19 between the 1st and 10th of December [8]. It is unclear whether the COVID-19 pneumonia related cases had occurred undetected in Wuhan, China prior to the 1st of December 2019 which requires further investigation.

Retrospective serological investigation of pneumonia cases in Wuhan before December 2019 will determine the extent of early unreported cases. It will also help determine whether SARS-CoV-2 circulated in Wuhan before December 2019 and will help track the origin of this outbreak among Chinese populations and humans in other parts of the world who had travel history to the epicentre prior to the known start of the outbreak. It was previously reported that sensitive and specific serological detection of MERS-CoV in subclinical infection is challenging [34,35]. SARS-CoV-2 can cause asymptomatic to fatal respiratory diseases [36]. Asymptomatic to mild SARS-CoV-2 infections can go unnoticed and there may be a lack of seroconversion or cross-reactivity in nucleic acid PCR-confirmed cases which requires further serosurveillance studies to help understand the antibody response of SARS-CoV-2 infections. Evaluation of the serologic response of SARS-CoV- 2 infected patients according to the disease severity will help determine the potential role of serodiagnostic parameters as prognostic markers. The development of accurate and robust serological assay will help determine the accurate SARS-CoV-2 prevalence.

Infections due to SARS-CoV-2 among healthcare workers and family clusters were also reported and human-to-human transmission has been confirmed [37], however further investigations are required to determine and understand the full extent of this mode of transmission. So far, there is no evidence of airborne transmission of the SARS-CoV-2, however precautionary measures are recommended due to the lack of information excluding this mode of transmission. The present COVID-19 outbreak is the third global alert of coronavirus infections. SARS-CoV-2 transmission in humans appears efficient and the virus is of pandemic potential. As of today, public health measures in China and certain affected areas are yet unable to halt the spread of human infections. There is great concern that spread of the virus may be devastating and of huge public health concerns globally, especially in resource-limited countries.

Based on the general definition of a pandemic as an infection that spreads globally, COVID-19 is already a “pandemic”. On January 30, 2020, the International Health Regulations Emergency Committee of the World Health Organization declared COVID-19 outbreak a public health emergency of international concern (PHEIC) [38]. Subsequently, the US declared it a public health emergency on January 31, 2020 [39], and several travel restrictions to the epicentre of the outbreak were imposed by the USA, Canada, UK, many countries in Europe, the Philippines, and several other countries have followed similar travel restrictions [40,41] to avoid SARS-CoV-2 infection importation by air travel. In addition, due to an ongoing COVID-19 outbreak in Italy and Iran in February 2020, several countriers in the Arabian Peninsula have imposed similar travel restrictions to affected areas. The current situation on COVID-19 confirmed cases in the Middle East and North Africa (MENA) countries is unclear and may be escalating and of great public health concern.

COVID-19 is a recent example of the complex threats of emerging infectious diseases. Emerging infections in humans and animals, along with other threats such as antimicrobial resistance, are difficult challenges to humanity, to a large extent driven by increasing food production and other issues related to a growing and more resource-demanding population. The interdisciplinary *One Health* approach represents an attempt to deal with such complex problems engaging professionals from many disciplines such as human, veterinary, and environmental health, as well as social sciences [42]. The One Health approach recognizes the interrelationship between animals, humans and the environment and encourages collaborative efforts to improve the health of people and animals, including pets, livestock, and wildlife [43]. One Health teams can work to identify sources of emerging pathogens and ways to reduce the threat of outbreaks [44]. The implementation and development of One Health collaborations on a global scale are critical to reduce the threats of emerging viruses [42,43].

Regarding SARS-CoV-2 in particular, there are several aspects that needs a One Health approach in order to understand the outbreak, and to mitigate further outbreaks of a similar virus. SARS-CoV-2 is likely a bat-origin coronavirus that was transmitted to humans through a spillover from bats or through yet undetermined intermediate animal host (avian, swine, phocine, bovine, canine, other species) or wild animals. Fig. 3 depicts a transmission hypothesis of SARS-CoV-2 outbreak, the potential intermediate host is yet to be determined. The list of animals which were sold in Huanan Seafood Market in Wuhan ranged from poultry (turkey, pheasants, geese, roosters, doves) wild birds (Peacocks, swans, others species), and exotic animals, to reptiles and hedgehogs. The animal list included frogs, camels, wild rabbits, reptiles, snakes, deer, crocodiles, kangaroos, snails, civet cats, goats, centipedes, and cicades [45,46]. There are no data available in scientific literature on the detection and isolation of SARS-CoV-2 from environmental samples. However, it was recently reported that the Chinese Centers for Disease Control and Prevention isolated SARS-CoV-2 from 33 samples out of 585 environmental samples collected from Huanan Seafood Market [47].

**Fig. 3:**
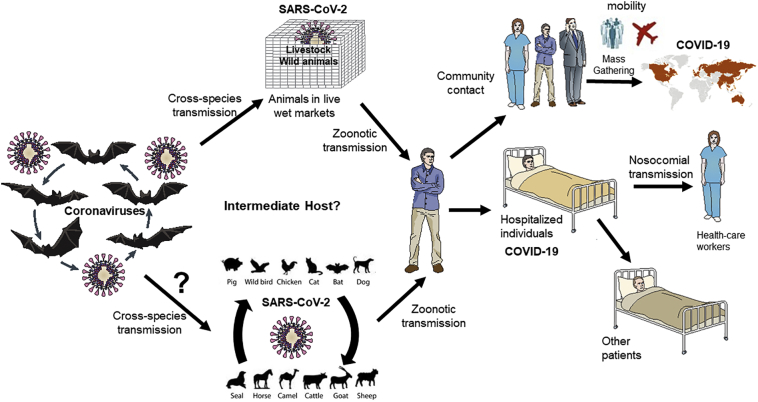
The emergence of SARS-CoV-2 and the outbreak of COVID-19. The figure depicts a hypothesized origin of the virus and a generalised route of transmission of the epidemic zoonotic coronavirus.

A broad surveillance for SARS-CoV-2 among different animals is warranted within Huanan Seafood Market and the vicinities, and should include potential reservoir hosts such as bats (both in the wild and if found in live animal markets) as well as potential intermediate hosts such as pigs, live poultry, fish, reptiles, and wild animals in close proximity to humans, which not only allow virus transmission among them but also lead to the generation of new viral strains. As a previous example, an HKU-2 bat origin, swine acute diarrhoea syndrome (SADS)-coronavirus emerged in the swine population in 2017 in Guangdong, China [27]. Surveillance for SARS-CoV-2 in nonhuman hosts including swine and wild animals and further genetic analysis of SARS-CoV-2, and other SARS-like CoVs may reveal the possible intermediate host of the recently emerged human SARS-CoV-2 responsible for the current outbreak and is a very important One Health measure. When the transmission chains and ecology of SARS-CoV-2 are clearer, the next step is to identify potential interventions to mitigate transmission. The unavailability of information on the possible intermediate host of SARS-CoV-2 and leaving the intermediate host undetermined are of high risk to humans and may well result in new outbreaks of SARS-CoV-2 or similar viruses, and future epidemics. In addition, exploring the intermediate host(s) of SARS-CoV-2 will help conduct further investigations to evaluate the host-pathogen relationship, disease dynamics and the possibility of reverse zoonosis. These interventions will likely include measures of several different types including several different disciplines [42,43,48]. Hypothesizing that bats will be proven as SARS-CoV-2 reservoirs and that adaptation to humans took place in an intermediate host in a live animal market, the following measures would be important:

i) Decreasing the risk of transmission from the natural host. This includes more knowledge of the natural ecology of the virus, so that high-risk transmission situations can be avoided. Also, it is important to consider transmission risks when new interfaces between bats and humans are created, e.g. when human habitations extend into bat habitats. Finally, there are important social science/behavioural measures conveying the message to the public which interactions with bats should be avoided (based on disease ecology knowledge).

ii) Decreasing the risk of transmission from the intermediate host. In principle, this risk could be avoided by completely separating bats from the intermediate host. Depending on which animal(s) is proven to constitute the intermediate hosts(s) for SARS-CoV-2, this may however be more or less difficult in practice. Likely, live animal markets play an important role in this process, and they need to be addressed in any true One Health approach [48]. However, it is crucial to consider the cultural context of these markets meaning that again, social sciences are important in this process. Also, this means that the most viable solution may not be to close down live animal markets but perhaps to ‘sector’ them so that fewer different species mingle in one specific market and that the specific intermediate host(s) for SARS-CoV-2 may be removed from the markets, or rigorously tested for the virus.

iii) Decreasing the human-to-human transmission. This is obviously a crucial measure to stop the current outbreak, and rightfully attracts the most attention at the present time. This review does not aspire to cover the large subject of human-to-human transmission control, but also here a mixture of measures is important from strictly medical (transmission routes, efficiency of PPE, vaccines, antivirals and so on) to more social science-oriented (How do people behave when they suspect they could be infected? How do they behave when they are sick? How to potentially change these behaviours?).

To successfully decrease the risk for a new SARS-CoV-2 outbreak or an outbreak of a similar virus, a One Health approach is crucial.

In conclusion, SARS-CoV-2 which causes COVID-19 is continuing to cause global fears, psychological distress, economic losses and negative impacts on several human activities including industry and mobility. To date, SARS-CoV-2 does not represent a pandemic threat with the same severity as e.g. the 1918 Spanish influenza, but could still cause a high number of deaths and put enormous strain on healthcare systems if widespread globally. Likely, resource-limited countries will be hit hardest due to smaller healthcare budgets and less possibilities of diagnostics and infection control.

There is an urgent need for the implementation of multidisciplinary One Health to address the current complex health challenges at the human-animal-environment interface [42,43].

One Health approaches in China have recently been described [[49], [50], [51], [52]]. However, the implementation of One Health policies in China is challenged by several barriers [48,51]. Should strict implementation of One Health measures in China have been implemented, the emergence of two coronaviruses (SARS in 2002–3 and SARS-CoV-2 in 2019) may have been prevented. Alarmingly, One Health policies are not yet implimented in several parts of the world where hotspots of infectious diseases are present which may result in potential emerging infections affecting humans. SARS-like coronaviruses of pandemic potential have been recently reported. [54,55]. Surprisingly, a recent study highlighted the risk of bat coronavirus outbreaks in China [56]. Therefore, further investigations using one health approaches will help predict virus hotspots and their cross-species transmission poential and the implimenation of one health policies are critical and urgently required.

The implementation of One Health measures in live animal markets in China (where SARS-CoV-2 is suspected to have emerged), will likely reduce the risk of emerging zoonotic viruses of pandemic potential in the future. These measures may include implementation of legislations but also collaborative interdisciplinary control measures between agricultural and public health sectors. Such measures include biosurveillance of live animal markets, improved biosecurity in livestock farms, live animal markets and during animal transportation, public education on zoonotic diseases, and the importance of adopting a cooperative approach between agencies.

The success in the containment of the current COVID-19 outbreak in China, affected countries, and the sporadic travel related cases worldwide will depend much on conventional public health measures, rapid clinical case identification, contact investigation, strict infection control in healthcare facilities, patient isolation, public education and community containment (quarantine) [53].

## Author statement

MEZ conceived the idea of the manuscript. MEZ wrote the initial draft, collected data, and generated fig. 4. JDJ revised the manuscript and contributed to writing and revisions. Both authors revised the final version of the manuscript.

## Funding

The Swedish Research Council (VR) grant number 2016-02606.

## Declaration of Competing Interest

We declare that we do not have any conflict of interest associated with manuscript. MEZ is a team member of Duke One Health, Duke University, Durham, North Carolina, USA.
